# Genome-wide tandem repeat expansions contribute to schizophrenia risk

**DOI:** 10.1038/s41380-022-01575-x

**Published:** 2022-05-12

**Authors:** Bahareh A. Mojarad, Worrawat Engchuan, Brett Trost, Ian Backstrom, Yue Yin, Bhooma Thiruvahindrapuram, Linda Pallotto, Aleksandra Mitina, Mahreen Khan, Giovanna Pellecchia, Bushra Haque, Keyi Guo, Tracy Heung, Gregory Costain, Stephen W. Scherer, Christian R. Marshall, Christopher E. Pearson, Anne S. Bassett, Ryan K. C. Yuen

**Affiliations:** 1 Genetics and Genome Biology, The Hospital for Sick Children, Toronto, ON Canada; 2 The Centre for Applied Genomics, The Hospital for Sick Children, Toronto, ON Canada; 3 Department of Molecular Genetics, University of Toronto, Toronto, ON Canada; 4 Clinical Genetics Research Program, Centre for Addiction and Mental Health, Toronto, ON Canada; 5 The Dalglish Family 22q Clinic for Adults with 22q11.2 Deletion Syndrome, Toronto General Hospital, University Health Network, Toronto, ON Canada; 6 Division of Clinical and Metabolic Genetics, The Hospital for Sick Children, Toronto, ON Canada; 7 McLaughlin Centre, University of Toronto, Toronto, ON Canada; 8 Genome Diagnostics, Department of Paediatric Laboratory Medicine, The Hospital for Sick Children, Department of Laboratory Medicine and Pathobiology, University of Toronto, Toronto, ON Canada; 9 Department of Psychiatry, University of Toronto, Toronto General Hospital Research Institute and Campbell Family Mental Health Research Institute, Toronto, ON Canada

**Keywords:** Genetics, Schizophrenia

## Abstract

Tandem repeat expansions (TREs) can cause neurological diseases but their impact in schizophrenia is unclear. Here we analyzed genome sequences of adults with schizophrenia and found that they have a higher burden of TREs that are near exons and rare in the general population, compared with non-psychiatric controls. These TREs are disproportionately found at loci known to be associated with schizophrenia from genome-wide association studies, in individuals with clinically-relevant genetic variants at other schizophrenia loci, and in families where multiple individuals have schizophrenia. We showed that rare TREs in schizophrenia may impact synaptic functions by disrupting the splicing process of their associated genes in a loss-of-function manner. Our findings support the involvement of genome-wide rare TREs in the polygenic nature of schizophrenia.

## Introduction

Schizophrenia is a major neuropsychiatric disorder with heritability estimated at ~79% [1]. Previous studies have revealed the role of copy number variants (CNVs) [2, 3] and small nucleotide variants in its pathogenesis [4]. However, those analyses were not designed to interrogate repetitive regions of the genome owing to challenges in detecting and interpreting such variation. In a separate study [5], we used a comprehensive analytic strategy to investigate tandem DNA repeats, which constitute ~6% of the human genome, and showed that genome-wide TREs are associated with the risk of autism spectrum disorder (ASD), a complex neurodevelopmental disorder with genetic risk that overlaps that of schizophrenia [6]. Our recent genome sequence analysis provided evidence supporting the involvement of TREs in schizophrenia through the identification of potentially damaging TREs in known disease-associated loci from a cohort of unrelated adults with schizophrenia [7].

Here, we used ExpansionHunter Denovo (EHdn) [8] and our established analytic approach to analyze TREs in the genomes of 257 unrelated adult cases with schizophrenia of European ancestry, 225 ancestry- and sequence-pipeline-matched individuals with no major neuropsychiatric disorders (non-psychiatric controls) [7], and in 2504 individuals from the 1000 Genomes Project [9] to estimate population frequency of TREs in cases and controls (*Methods*). Our study was driven by the historical observation of anticipation in schizophrenia [10], and the fact that most of the known tandem repeat disorders are caused by rare TREs [11, 12]. Therefore, we specifically assessed for large rare TREs in schizophrenia. Our approach interrogates the entire genome irrespective of prior knowledge of the presence or expected sequence of tandem repeats in any given region, and focuses on tandem repeats having motifs of 2–20 bp for which the total repeat tract length is greater than the sequencing read length (i.e., >150 bp) [8]. We define a tandem repeat to be expanded when its tract length is an outlier compared to lengths at that loci in other individuals [5] (*Methods*).

## Materials and methods

### Ethics statement

This study was approved by the Research Ethics Board at the Centre for Addiction and Mental Health (CAMH) (151/2002-02) and other local REBs. Written informed consent was obtained for all participants [7].

### Samples sequencing, and genome alignment

We used genome sequencing data from Canadian individuals of European descent (257 with schizophrenia, and 225 with congenital heart disease (CHD) and no psychotic illness), as well as 2504 samples from the 1000 Genomes Project (1000G) [9]. The schizophrenia samples and non-psychiatric controls were assessed for quality, and prepared using TruSeq DNA library prep kits. These samples were sequenced on the Illumina HiSeq X platform (2 × 150 bp paired-end reads) at The Centre for Applied Genomics (TCAG, Toronto, Canada) and processed for alignment and genomic variant calling as previously described [7, 13]. The 1000 G samples were sequenced on the Illumina NovaSeq platform (2 × 150 bp paired-end reads). The 1000 G genome sequencing data are publicly available and we downloaded them via Amazon Web Services (s3://1000genomes/1000G_2504_high_coverage/data). All samples were aligned to the GRCh38/hg38 reference genome using BWA-mem [14]. The study protocol was approved by the Research Ethics Boards of The Hospital for Sick Children and CAMH. Informed consent was obtained from all participants at the recruitment locations.

### Genome-wide identification of tandem repeats

Genome-wide detection of tandem repeats was performed as previously described [5]. Briefly, we used ExpansionHunter Denovo (EHdn; https://github.com/Illumina/ExpansionHunterDenovo) [8] to estimate the size and location of genomic tandem repeats. For a tandem repeat to be detected by EHdn, it must be larger than the sequence read length (for example, >150 bp). As a result, samples that did not meet this minimum size for a given region were left without size estimation by EHdn. EHdn estimates the size of a tandem repeat by counting the number of anchored in-repeat reads (IRRs), which are read pairs in which the first read (the IRR) contains repetitive sequence and the second read (the anchor) contains non-repetitive sequence that can be uniquely mapped to the reference genome, thus allowing the repeat’s location to be determined. Although the EHdn sizes cannot be interpreted as exact numbers of base pairs, they are proportional to the number of base pairs comprising the repeat (see Fig. 1 of ref. [8]). To account for samples with different overall depths of coverage, the anchored IRR counts are normalized by the overall read depth of a given sample. We compared the tandem repeats identified by EHdn to tandem repeats in the human reference genome, derived from Tandem Repeats Finder (TRF) [15]. To support the accuracy of EHdn-predicted tandem repeat sizes, we genotyped the 8 rare exon-proximal repeat loci (9 motifs) identified in 13 individuals, along with 6 selected rare intronic repeat loci, using ExpansionHunter v.3.0.2 [16, 17], which estimates allele-specific tandem repeat sizes for each genomic coordinate and motif supplied by the user with high accuracy (precision = 0.91, recall = 0.99) [16, 17]. EHdn has been shown to be both sensitive (i.e., successfully rediscovered TREs in several disease-associated genes and was able to detect 77% of repeats >150 bp discovered by long-read sequencing [8], and specific (TREs that EHdn detects have been validated using orthogonal methods in several different studies [5, 7, 8, 18, 19]).

**Fig. 1:**
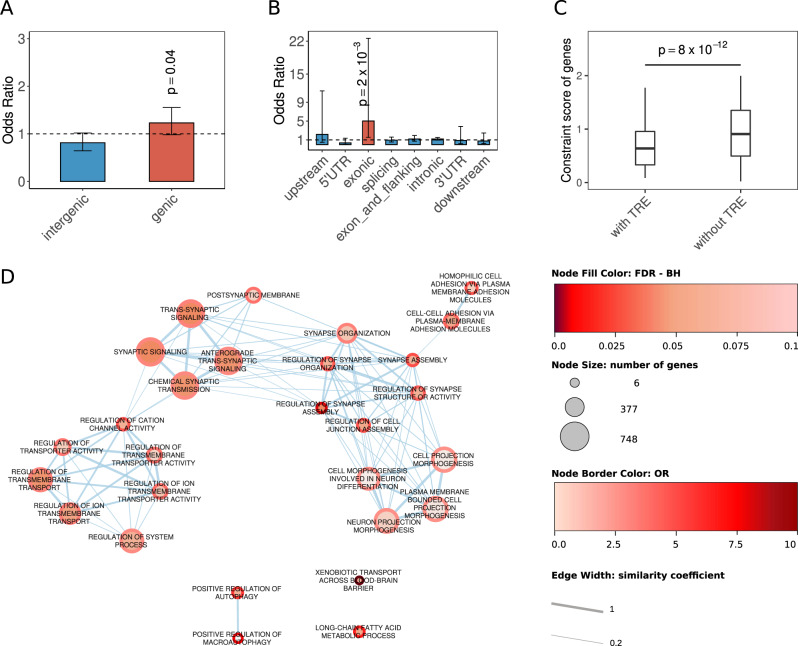
Genetic features and functional impact of rare TREs. **A** Burden analysis of rare TREs located near (genic) or outside (intergenic) genes. **B** Burden analysis of rare TREs with different genomic features in individuals with schizophrenia relative to non-psychiatric controls. Red bar indicates significant enrichment of exon-proximal (labelled as exonic) rare TREs in individuals with schizophrenia. Horizontal dashed line represents odds ratio = 1. **C** Distributions of gnomAD observed/expected (o/e) upper bound LOEUF values are shown for 182 genes with rare TREs (182 of 193 TRE-associated genes have scores) in the 220 (of 257) individuals with schizophrenia, compared with 18,990 genes with no such TREs identified in the schizophrenia cohort (one-sided Wilcoxon rank-sum test). Minima and maxima indicate 3× the interquartile range-deviated o/e upper bounds from the median and the centre indicates the median of the o/e upper bound values. **D** A map of gene functions enriched in genes associated with rare TREs. Each node represents a gene-set with its size proportional to the number of genes involved. The significant enrichment of a particular function was assessed by one-sided Fisher’s exact test. The false discovery rate and odds ratio represented by the color gradient and the width of edge is proportional to the similarity coefficient between gene functions. Synaptic mechanisms prevail.

To determine more precise coordinates for input to ExpansionHunter, we identified coordinates from TRF that overlapped each locus. For each combination of TRF coordinates and EHdn motif, we used ExpansionHunter to estimate motif-specific (as detected by EHdn) tandem repeat sizes for the samples involved. We then calculated the Spearman correlation coefficient and *P* value between the EHdn-predicted tandem repeat sizes and the size estimated by ExpansionHunter (defined as either the size of the longest allele or the sum of the two allele sizes), aggregated over all of the EHdn-detected motifs for that locus (Supplementary Table S7). We also manually evaluated the presence of tandem repeat expansions (TREs) and the corresponding motif by inspecting reads from the BAM file for tandem repeats that were found to be expanded by EHdn for all.

### Detection of rare tandem repeat expansions and sample quality assessment

We excluded tandem repeats with different size distribution between schizophrenia and CHD samples (two-sided Wilcoxon’s signed-rank test *P* < 0.05) in order to avoid any potential technical biases in estimating tandem repeat size between the two cohorts. To detect rare TREs, we followed Trost et al. [5] by applying the non-parametric Density-Based Spatial Clustering of Applications with Noise (DBSCAN) algorithm to identify outliers based on EHdn estimated tandem repeat size at each locus. We optimized two DBSCAN parameters as well as the population frequency cut-off for rare TRE identification (Supplementary Fig. S3; Supplementary Table S8). Based on a different set of rare TREs identified using different DBSCAN parameters and population frequency cut-off, burden test of exonic TREs and intergenic TREs were performed with the total number of rare TREs as a covariate. The DBSCAN parameters set (minclust = 11, eps = 2 × mode of EHdn sizes) and population frequency cut-off (frequency < 0.05) that provided the strongest signal in the exonic TREs burden test and weakest signal in intergenic TREs burden test was selected for the next step of the analysis. Sample quality assessment was done by inspecting the counts of total number of tandem repeat loci and TREs per sample. Anscombe transformation was done on the counts to put the count distribution closer to the gaussian distribution. Three control samples and five schizophrenia samples with the transformed counts exceeding 3 standard deviations from the mean of the transformed counts were tagged as outliers and excluded from the analysis. Rare TRE identification was then performed on the remaining set.

### Burden analysis

To compare the prevalence of rare TREs in individuals with and without schizophrenia, we performed a logistic regression analysis by regressing the number of rare TREs on the affected status (unaffected = 0, affected = 1). For this analysis, we only included tandem repeats on autosomal chromosomes to avoid sex bias. Biological sex and the total number of rare TREs per individual were used as covariates. To test the burden of TREs in different functional elements (for example, exons and introns), we separated the genome (RefSeq, GRCh38) into different functional elements: upstream (1 kb upstream of the transcriptional start site, TSS), 5′ untranslated regions (5′ UTR), exon, core splice site, intron, 3′ UTR and downstream (1 kb downstream of transcription termination sites) [4]. If any rare TRE affected more than one functional element, we prioritized the effects based on their impact on the corresponding genes predicted by ANNOVAR (October 2019 release) [20]. One-sided Wald test was performed, assuming a higher burden of rare TREs in cases with schizophrenia. Empirical *p* values from 10,000 case-control label permutations were reported.

### Experimental validation of tandem repeat expansions

Validation of the *SHANK1* TREs detected by EHdn was completed by PCR, gel electrophoresis, and Sanger sequencing. We designed primers flanking the repeat of interest with the following sequences: 5′-CCTATCTCCTATGAATGGACGAC-3′ and 5′-GATGCCGTTAAATGCGAGTTTC-3′. We performed PCR on the samples using HotStarTaq DNA polymerase (Qiagen), a primer annealing temperature of 63 °C, and an elongation time of 2 min. We then ran the PCR products on a 1.2% agarose gel to confirm the size of the repeats, and performed Sanger sequencing to confirm the sequence. Other DNA samples from this cohort without a predicted expansion in *SHANK1* were run under the same conditions as negative controls (Supplementary Fig. S2).

Validation of the *DAB1* TREs detected by EHdn was completed by PCR, gel electrophoresis, and Sanger sequencing. Primers flanking the repeat of interest were used with the following sequences: 5′-ATTTGCCCTTTGCTGATTGA-3′ and 5′-TGAAACTGAGGCTCAAAATGA-3′ [21]. We performed PCR on the samples using PrimeSTAR GXL DNA Polymerase (Takara), a primer annealing temperature of 61 °C, and an elongation time of 2 min. We then ran the PCR products on a 1.2% agarose gel to confirm the size of the repeats, and performed Sanger sequencing to confirm the correct region was amplified. Other DNA samples from this cohort without a predicted expansion in *DAB1* were run under the same conditions as negative controls.

### Statistical comparison of clinical features

**Fig. 2:**
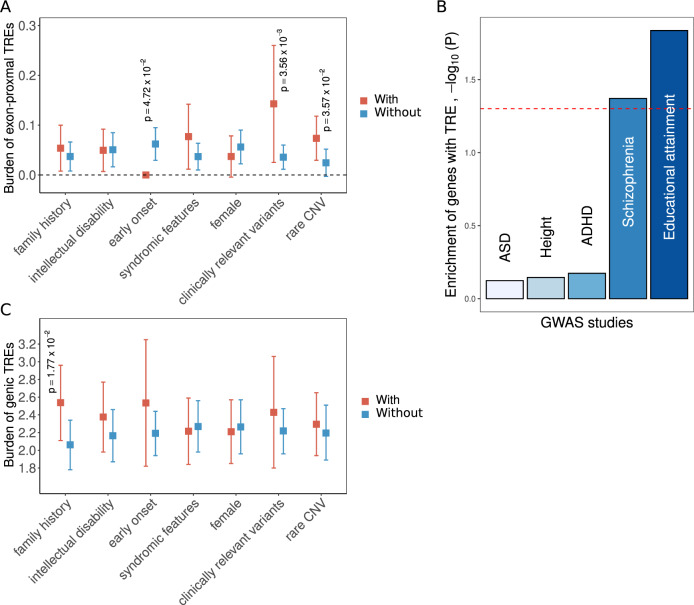
Genetic and clinical features involved in rare genic TREs in schizophrenia. The burden of (**A**) rare exon-proximal and (**C**) rare genic TREs in schizophrenia was analyzed with respect to presence/absence of seven variables (*x*-axis). Orange and blue colored boxes indicate results for TRE-containing individuals, with and without each of the seven variables, respectively, with vertical bars representing 95% confidence intervals; *p* values are provided above variables noting significant differences. No individuals with a rare exon-proximal TRE had an early age at onset of schizophrenia. **B** MAGMA was used to assess the 193 genes with rare TREs detected by our pipeline against proximity (<10 kb) to common risk variants from GWAS studies for schizophrenia, autism spectrum disorder (ASD), attention deficit/hyperactivity disorder (ADHD), educational attainment, and (as a negative control) height (*Methods*). The dashed red line represents association with *p* value equal to 0.05.

We hypothesized that individuals with schizophrenia compared with CHD and no psychotic illness (Fig. 1A, B), and differentially within schizophrenia those with clinical features, including family history of schizophrenia in first degree relatives, intellectual disability and syndromic features (Fig. 2A and 2C), would have a greater contribution from the genic and exonic (i.e., exon-proximal, within 300 bp of exon junctions) TREs identified. Therefore, we used the non-parametric one-sided Wilcoxon signed-rank test to compare the datasets unless stated otherwise.

### Enrichment in common variant risk

For the 193 TRE-associated genes that we identified in this schizophrenia sample, we used MAGMA v.1.09b11 as described previously [22] to determine whether they were enriched in common variant risk loci for schizophrenia and other traits. Specifically, we compared our 193 TRE-associated gene set against summary statistics from genome-wide association studies (GWASs) for schizophrenia [23], ASD [24], attention deficit/hyperactivity disorder [25], educational attainment [26] and (as a negative control) height [27] (Fig. 2B, Supplementary Table S9). We also tested for an enrichment of 193 TRE-associated genes in the 655 genes within 270 refined genome-wide significant loci that involve fewer than 4 causal variants in the latest schizophrenia GWAS of 69,369 cases and 236,642 controls [14]. We applied one-sided Fisher’s Exact test to compare the enrichment of GWAS signals in TRE-associated genes (*n* = 193) against other genes that are not associated with rare TRE (*n* = 19,276).

### Functional enrichment analysis

For the functional enrichment test, we used a one-sided Fisher’s exact test and gene ontology terms (from the R Bioconductor library org.Hs.eg.db v3.13.0), restricting the sets to those with a number of annotated genes between 5 and 1000. The *p* values were then corrected for multiple comparisons with the Benjamini Hochberg false discovery rate procedure. The results were loaded in Cytoscape v3.8.1 with the Enrichment map plugin v3.3.3 [28], filtering out sets with false discovery rate >0.1 and similarity coefficient (combined coefficient *k* = 0.5) lower than 0.2, to retain only the most significant terms.

## Results

After quality assessment and parameter optimization (*Methods*), we performed a burden analysis comparing rare TREs (<0.5% frequency in 1000 Genomes Project individuals) in individuals with schizophrenia and in non-psychiatric controls. We identified 583 rare TREs in 436 distinct regions in 220 individuals with schizophrenia (Supplementary Table S1); 199 of these distinct regions were genic (involving 193 genes, hereafter referred to as TRE-associated genes, including 6 genes with multiple repeat motifs/regions identified). In individuals with schizophrenia, rare TREs tend to be located within genes (*p* = 0.04, Fig. 1A), and more likely to be at exon junctions (odds ratio (OR) = 5.03, *p* = 2 × 10^−3^, Fig. 1B). Fine mapping of the eight exon-proximal TREs revealed their precise locations to be in intronic or untranslated regions with close proximity (<300 bp) to protein-coding exons. This included a CTG expansion in myotonic dystrophy-linked *DMPK* we reported previously [7] (Supplementary Information and Supplementary Table S2). The proportion of schizophrenia cases with at least one rare exon-proximal TRE is 5.16%, while the proportion of controls with at least one rare one exon-proximal TRE, after correcting for bias in the intergenic region, is 1.20%. Thus, we estimate from this study that the rare exon-proximal TREs may collectively account for 3.96% of the risk in schizophrenia.

In 139 of the individuals with schizophrenia, there were 222 rare intronic TREs in 160 distinct regions of 155 genes (including 5 genes with multiple repeat motifs/loci), representing the largest subcategory of the genic region. Of these, 51 individuals had rare intronic TREs in one or more of the 38 genes that are associated with neurological abnormality, abnormal behavior or nervous system abnormality in Mammalian Phenotype Ontology, including genes previously associated with schizophrenia such as *DCLK1*, *ERBB4*, *GRIK4*, *GRIN2A*, *SHANK1*, and *VIPR2* (Supplementary Table S3). Gene-set analysis of rare exon-proximal and intronic TREs identified a significant enrichment of genes involved in postnatal brain expression, such as *GRIN2A* and *SHANK1* (OR = 1.83, *p* = 8.6 × 10^−3^, false discovery rate = 0.2) (Supplementary Table S4 and Supplementary Fig. 1).

The motifs involved in the TREs are diverse in terms of size and sequence (Supplementary Table S1). The GC content of the involved motifs of the rare TREs studied is significantly lower than in the unexpanded tandem repeats, or in the known pathogenic repeats, but is higher than that found in ASD (Supplementary Fig. S2). TRE-associated genes were significantly more constrained than other genes as measured by the GnomAD loss-of-function observed/expected upper bound fraction (LOEUF) [29] (*p* = 3 × 10^−10^) (Fig. 1C). Rare TREs are found more frequently in regions that are closer to the splice junction (Supplementary Fig. 3), and the TRE-associated genes are predominantly involved in synaptic functions and signaling pathways (Fig. 1D), which are commonly known to play a role in brain development and believed to be involved in the etiology of schizophrenia [2, 4, 30, 31]. Collectively, these findings suggest that the rare TREs in schizophrenia may impact synaptic functions by disrupting the splicing process of their associated genes in a loss-of-function manner.

Our previous analysis of this same community-based cohort of adults with schizophrenia included 33 individuals with clinically relevant CNVs and small nucleotide variants, constituting 12.8% of individuals studied [7, 32, 33]. Of the 13 individuals with the eight rare exon-proximal TREs, five had at least one other clinically relevant (non-TRE) rare variant (i.e., small nucleotide/CNVs) (Supplementary Table S5); [7, 32, 33] a significant association (*p* = 3.56 × 10^−3^) (Fig. 2A, and Supplementary Information). This supports schizophrenia as a complex disorder involving multiple genetic risk factors [30].

Next, we used MAGMA [22] to integrate summary statistics from GWASs of five traits (ASD, height, attention deficit hyperactivity disorder, schizophrenia, and educational attainment), and examined whether common genetic variation influencing these traits were located within 10 kb of the 193 TRE-associated genes we identified (*Methods*). We determined that signals for schizophrenia and for educational attainment (where the GWAS signal also correlates with schizophrenia), but not the other three GWAS signals tested (*Methods*), showed significant enrichment for our TRE-associated genes (Fig. 2B), further supporting their contribution to the polygenic risk of schizophrenia. Eleven of the 193 TRE-associated genes, including *DPYD* and *EFNA5*, were disproportionally found amongst the 655 genes potentially tagged by genome-wide significant common variant signals at 270 loci in the latest schizophrenia GWAS (OR = 2.65, *p* = 5 × 10^−3^), but only two (*GRIN2A* and *MYT1L*) were in the 114 protein-coding genes prioritized using current variant mapping and expression methods [31].

In our schizophrenia cohort, we also found that individuals with a family history of schizophrenia were more likely to carry rare TREs in genic regions (*p* = 1.77 × 10^−2^) (Fig. 2C), suggesting that these individuals may have inherited the rare TREs. To investigate the involvement of rare genic TREs in families with a history of schizophrenia, we performed genome sequencing on 63 additional individuals (30 of whom were diagnosed with schizophrenia) from 14 independent families with an extended family history of schizophrenia [34]. In two probands of these 14 families, we detected rare TREs in three genic regions (*CALCOCO2* and *FXN* in one family, and *SHANK1* in the other family) that were also identified in our primary schizophrenia cohort (Supplementary Table S6). In these two families, all five genome-sequenced individuals with schizophrenia carried the detected rare genic TREs, even though the same expansions can be found in some of the unaffected family members (Supplementary Table S6). These include two individuals with the AAAAG expansion in *SHANK1* that targeted genotyping delineated to have originated from the paternal side of the family (Supplementary Fig. S4). These findings further substantiate the contribution of rare TREs to the heritable risk of schizophrenia.

Across both the original and familial cohorts, we identified and validated three known disease-associated tandem repeats. In the family that has rare TREs in *FXN*, individual III-1 has an expanded repeat size in *FXN* in the pathogenic range for Friedreich ataxia, and about three times larger on average than those detected in the individuals from the previous generation, with a diagnosis of this autosomal recessive disorder confirmed in clinical records (Supplementary Table S6). In the unrelated primary schizophrenia cohort, we previously reported a > 200 repeat-long CTG expansion in *DMPK*, within the known pathological range, and consistent with a history of myotonic dystrophy in the individual’s family [7]. Also, for the three unrelated individuals with rare intronic TREs in *DAB1* (Supplementary Table S3), encoding a reelin adaptor protein, we confirmed the sizes of expanded ATTTT repeats (Supplementary Fig. S5), which are comparable to rare TREs reported by others at this locus for spinocerebellar ataxia type 37 [21]. Consistent with having no diagnosis, or clinical signs, of spinocerebellar ataxia, however, the three individuals in our study had no ATTTC repeat insertions in the expanded repeat tract in *DAB1* [21].

## Discussion

We demonstrate that rare TREs, in particular those that are intronic and close to exons, are an important class of variants contributing to the etiology of schizophrenia. The functional and constraint profiles of the implicated genes, the proximity of these genes to GWAS signals for schizophrenia, and the proximity of the rare repeats to coding sequence and to splice junctions, are consistent with the relevance of rare intronic and exon-proximal TREs to schizophrenia-related mechanisms. We estimate from this study that the rare exon-proximal TREs may collectively account for 3.96% of the risk in schizophrenia.

While epigenetic modifications are known as a gene-disrupting mechanism in some well-known TREs [12, 35], such as CGG repeat expansions in *FMR1*, we found that CG-containing motifs are uncommon in the tandem repeats expanded in schizophrenia (Supplementary Table S1), and in fact are significantly less common than the unexpanded tandem repeats or the known pathogenic repeats (Supplementary Fig. S2). This suggests that epigenetic modifications are unlikely to represent the main mechanism involved in schizophrenia. Instead, our results show that rare TREs in schizophrenia differentially impact synaptic functions (Fig. 1D), and that the mechanism is likely to involve disrupting the splicing process of their associated genes in a loss-of-function manner (Fig. 1C, Supplementary Fig. S3).

One example of the synaptic genes recurrently affected by rare TREs is *SHANK1*, a postnatal brain-expressed gene that encodes scaffold proteins that are required for the development and function of neuronal synapses [36]. Genetic variants, including rare CNVs encompassing *SHANK1*, have been observed in individuals with non-syndromic ASD [37, 38] (Supplementary Information). Further studies are warranted to delineate the mechanisms of TREs in regulating the expression of *SHANK1*, and for the other TRE-associated genes identified, during brain development.

Family history of schizophrenia was the only clinical factor assessed that was significantly associated with rare genic TREs (Fig. 2C). This may be consistent with inheritance of TREs and with the historical observation of clinical anticipation in familial schizophrenia [10]. Notably, a higher degree of polygenic risk for schizophrenia is also associated with positive family history [7]. As for most studies of genetic variants in schizophrenia, there was no association of rare TREs with age at onset. Consistent with findings for other rare variants associated with schizophrenia, and with incomplete penetrance, we found the same TREs to be present in some of the unaffected members in our family studies (Supplementary Table S6). For individuals with clinically relevant variants, or with rare CNVs who a priori were deliberately oversampled in this cohort [7], there was elevated burden of rare exon-proximal TREs (Fig. 2A), suggesting the possibility that rare TREs may act additively to increase the risk of schizophrenia.

Our approach may have increased the power to detect the rare TREs that collectively contribute to schizophrenia in a relatively small sample. We chose an unbiased genome-wide assessment of large rare TREs (>150 bp). Short de novo tandem-repeat expansions and contractions (e.g., of repeat size ≤150 bp) may impose additional schizophrenia risk [39]. These can be detected from sequence data by standard small variant calling algorithms as small insertion/deletions (i.e., indels), thus their contribution could well have been captured by previous exome or genome sequencing studies of schizophrenia [4, 40]. Complementary designs, including studies with a much larger sample size, are required to assess variants likely to have small effect sizes, such as common tandem repeats.

Due to the rarity of the TREs studied, determining the penetrance for individual expanded loci is impossible in this study. However, some of the rare TREs identified, such as the CTG repeat expansion in *DMPK*, have also been found in ASD [5]. This suggests a pleiotropic effect of TREs, which is consistent with many other schizophrenia-associated genetic variants [2, 40]. Further characterization of their effects and inheritance across large cohorts of multiple neuro-psychiatric/developmental disorders, and across generations within families, may help resolve the pleiotropic effects and penetrance for individual TREs. Future studies should also examine the potential impact of somatic TREs, which we have not assessed here due to the limitations of existing algorithms, and the use of blood, not brain, samples.

Involvement of genome-wide TREs in schizophrenia may help explain the clinical genetic anticipation that has long been recognized in schizophrenia and suspected to be related to tandem repeats, with the additional possibility that multiple risk variants accumulate over generations [10, 41]. The current study adds to support for both possibilities. The enrichment of common variant signals for schizophrenia GWAS at the TRE-associated genes identified further supports TRE contributions to the genetic architecture of schizophrenia. Our findings suggest rare TRE as a potential source of some of the missing heritability for schizophrenia, and highlight the necessity of further genome sequencing studies of TREs in other complex disorders for which missing heritability remains to be identified [42].

## Supplementary information

Supplementary information and figures

Supplementary Tables

## Data Availability

The 1000G genome-sequencing data are publicly available via Amazon Web Services (s3://1000genomes/1000G_2504_high_coverage/data).

## Code availability

Code used in this paper is available from the corresponding author upon reasonable request.
