# Supplementary material for: Genome-wide tandem repeat expansions contribute to schizophrenia risk
Source: Mol Psychiatry. 2022 May 12;27(9):3692–8. doi: 10.1038/s41380-022-01575-x (PMC9708556; doi:10.1038/s41380-022-01575-x)
Supplement: Supplementary file 1 — Supplementary information and figures [file 41380_2022_1575_MOESM1_ESM.pdf]

Genome-wide tandem repeat expansions contribute to schizophrenia risk

Bahareh A Mojarad[1], Worrawat Engchuan[1,2], Brett Trost[1,2], Ian Backstrom[1], Yue Yin[1], Bhooma Thiruvahindrapuram[1,2], Linda Pallotto[1], Aleksandra Mitina[1], Mahreen Khan[1,3], Giovanna Pellecchia[1,2], Bushra Haque[1,3], Keyi Guo[1], Tracy Heung[4,5], Gregory Costain[1,3,6], Stephen W Scherer[1,2,3,7], Christian R Marshall[8], Christopher E Pearson[1,3], Anne S Bassett[4,5,9*], Ryan KC Yuen[1,3*]

[1]Genetics and Genome Biology, The Hospital for Sick Children, Toronto, Ontario, Canada
[2]The Centre for Applied Genomics, The Hospital for Sick Children, Toronto, Ontario, Canada
[3]Department of Molecular Genetics, University of Toronto, Toronto, Ontario, Canada
[4]Clinical Genetics Research Program, Centre for Addiction and Mental Health, Toronto, Ontario, Canada
[5]The Dalglish Family 22q Clinic for Adults with 22q11.2 Deletion Syndrome, Toronto General Hospital, University Health Network, Toronto, Ontario, Canada
[6]Division of Clinical and Metabolic Genetics, The Hospital for Sick Children, Toronto, Ontario, Canada
[7]McLaughlin Centre, University of Toronto, Toronto, Ontario, Canada
[8]Genome Diagnostics, Department of Paediatric Laboratory Medicine, The Hospital for Sick Children, Department of Laboratory Medicine and Pathobiology, University of Toronto, Toronto, Ontario, Canada
[9]Department of Psychiatry, University of Toronto, Toronto General Hospital Research Institute and Campbell Family Mental Health Research Institute, Toronto, Ontario, Canada
[*]These authors contributed equally
Corresponding author: Ryan KC Yuen ryan.yuen@sickkids.ca

Supplementary information

Clinically relevant rare variants in individuals with schizophrenia:
Clinically relevant rare variants were defined as small nucleotide changes (single nucleotide variants, and small insertions/deletions) and copy number variants (CNVs) classified as pathogenic/likely pathogenic as potentially clinically relevant and contributing to the expression of schizophrenia, following the guidelines provided by the American College of Medical Genetics and Genomics (ACMG)[1,2] . For complete information on the clinically relevant rare variants please refer to Mojarad et al[3].

(CTG)n expansions in 3'UTR of *DMPK* and schizophrenia:
We have previously reported CTG expansions in myotonic dystrophy (DM1)-linked *DMPK* in individuals with schizophrenia and with autism spectrum disorder (ASD)[3,4]. Interestingly, individuals with DM1 had also previously been reported to show signs of schizophrenia, cognitive impairement, social and personality disorders[5-8]. Transgenic mice with >1000 CTG units in *DMPK* show RNA toxicity in the brain, and exhibit neurochemical and electrophysiological signs of synaptic dysfunction, as well as behavioral and cognitive phenotypes[9].

Supplementary Figures

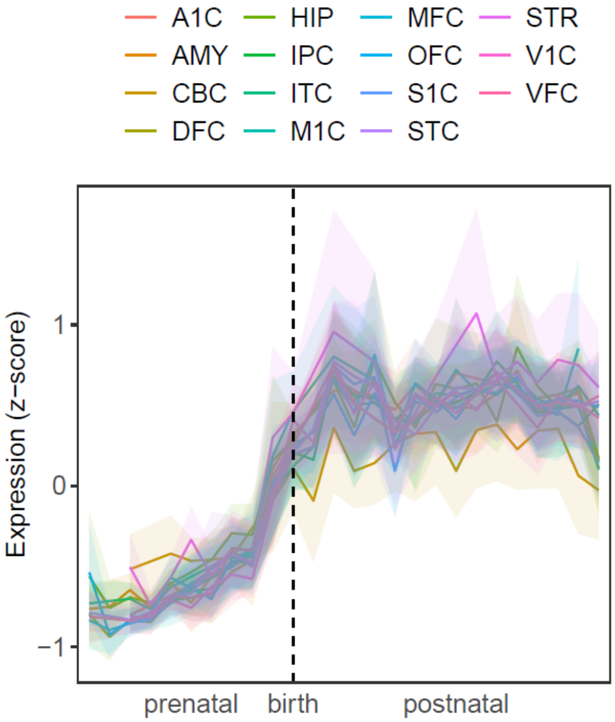

Supplementary Figure S1. Temporal expression of genes with intronic TREs in different regions of human brain. Expression in different regions of human brain throughout developmental stages of the postnatally expressed genes (a significantly enriched gene-set, Supplementary Table S4) with intronic TREs in our schizophrenia cohort (Supplementary Table S3). Gene expression for each gene with available data was obtained from the BrainSpan Allen Brain Atlas (http://www.brain-map.org) of Developing Human Brain. Each graph line represents the average expression of 35 genes in each of 15 different brain regions and respective shaded areas represent the 95% confidence intervals of the expression. The results show the intronic TRE gene expression pattern across multiple brain regions.

A1C: primary auditory cortex (core); AMY: amygdaloid complex; CBC: cerebellar cortex; DFC: dorsolateral prefrontal cortex; HIP: hippocampus (hippocampal formation); IPC: posteroventral (inferior) parietal cortex; ITC: inferolateral temporal cortex (area TEv, area 20); M1C: primary motor cortex (area M1, area 4); MFC: anterior (rostral) cingulate (medial prefrontal) cortex; OFC: orbital frontal cortex; S1C: primary somatosensory cortex (area S1, areas 3,1,2); STC: posterior (caudal) superior temporal cortex (area 22c); STR: striatum; V1C: primary visual cortex (striate cortex, area V1/17); VFC: ventrolateral prefrontal cortex.

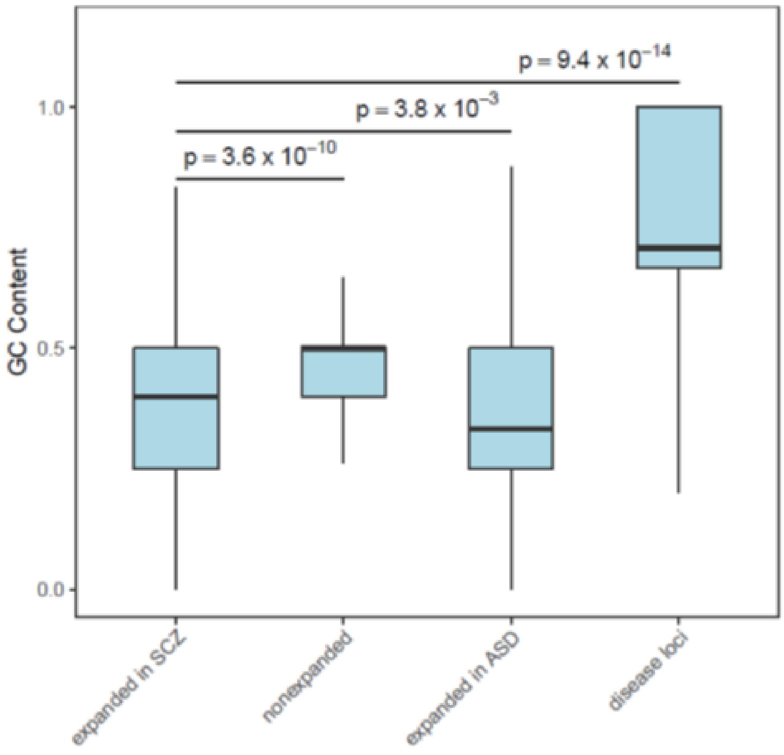

Supplementary Figure S2. Comparison of GC content in the motifs of TREs. The GC content of motifs in tandem repeats that are expanded in schizophrenia (expanded in SCZ) is compared to those i) that are found in nonexpanded tandem repeats in schizophrenia (nonexpanded), ii) that are expanded in autism spectrum disorder (expanded in ASD), and iii) that are associated with known tandem-repeat disease loci (disease loci). The known tandem-repeat disease loci can be found in Table 1 in Gall-Duncan et al[10].

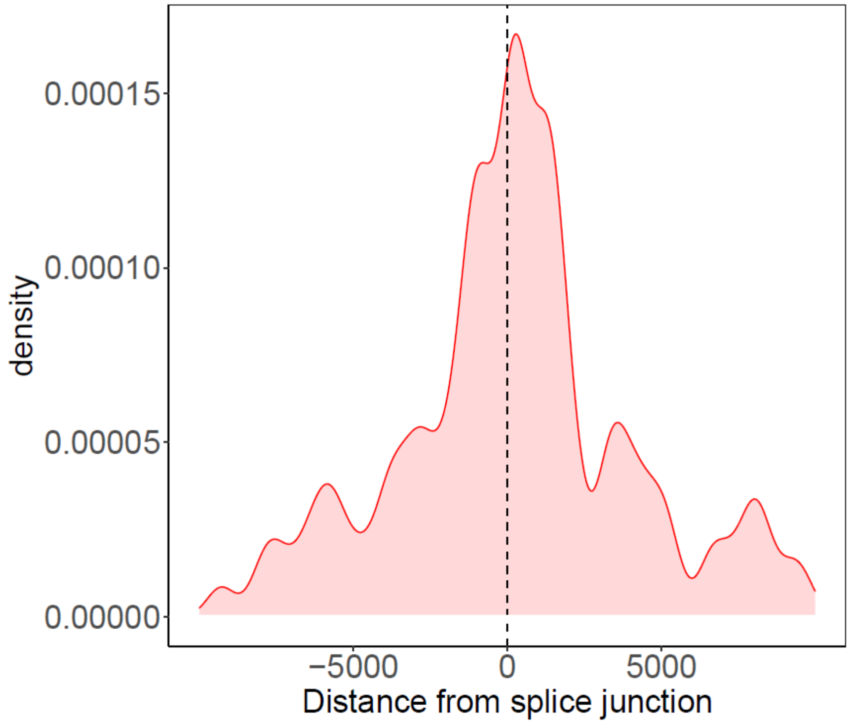

Supplementary Figure S3. Distance to the nearest splice junction of all rare tandem repeat expansions identified in the schizophrenia cohort. The density represents the frequency of a rare tandem repeat expansion found at a specific distance from the nearest splice junction.

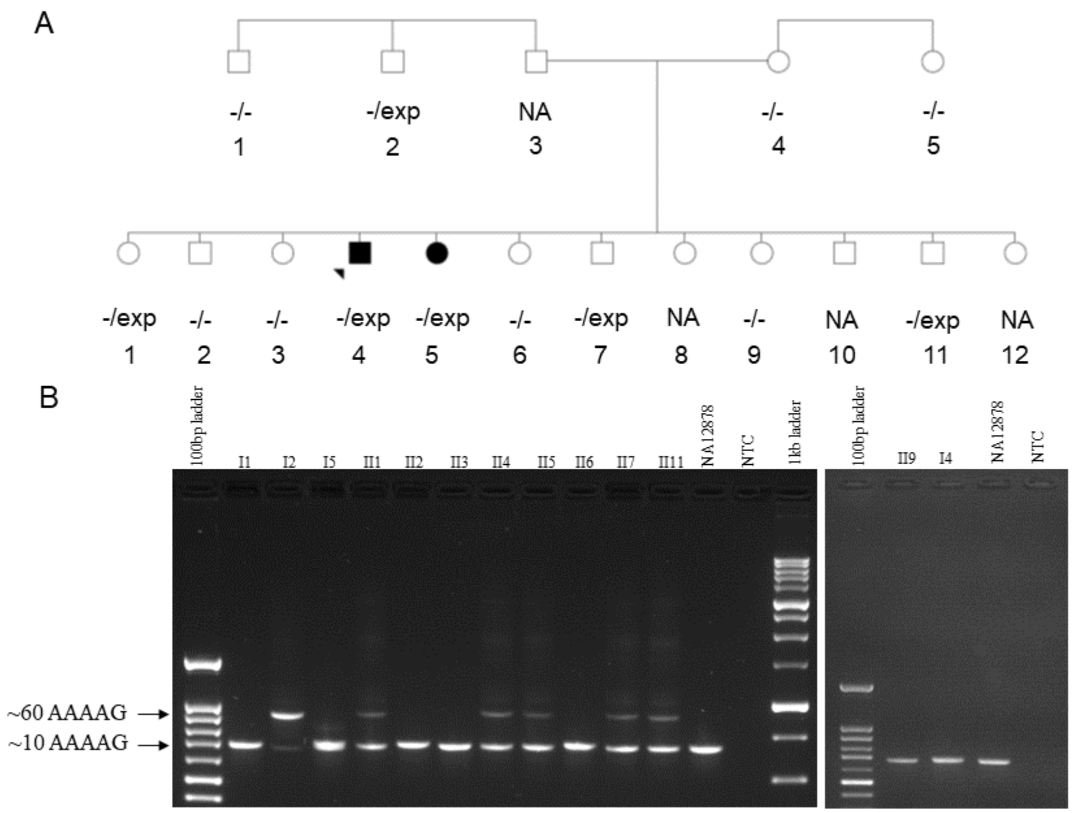

Supplementary Figure S4. An intronic tandem DNA repeat in *SHANK1* is expanded in an extended family with schizophrenia. (**A**) Pedigree of family 2 with an (AAAAG)n tandem repeat expansion detected in an intron of *SHANK1* (also identified in an unrelated proband with schizophrenia from the main cohort, Supplementary Tables S1 and S3). Expansions were originally detected using ExpansionHunter Denovo on II-4 (proband) and II-11 (unaffected sibling), whose DNA was genome-sequenced (Supplementary Table S6). Presence of the *SHANK1* intronic expansion (exp) was then assessed by targeted PCR assay for eleven other individuals in the family (Family 2). (**B**) The gel electrophoresis showing one amplicon size (~300bp/~60 additional repeats) larger than those detected in the control DNA (NA12878) corresponding to the intronic TREs identified in *SHANK1* in six of the thirteen individuals (including II-4 and II-5 with schizophrenia) from Family 2; DNA was extracted from peripheral blood or lymphoblast cell lines. NA: not available. NTC: No template control.

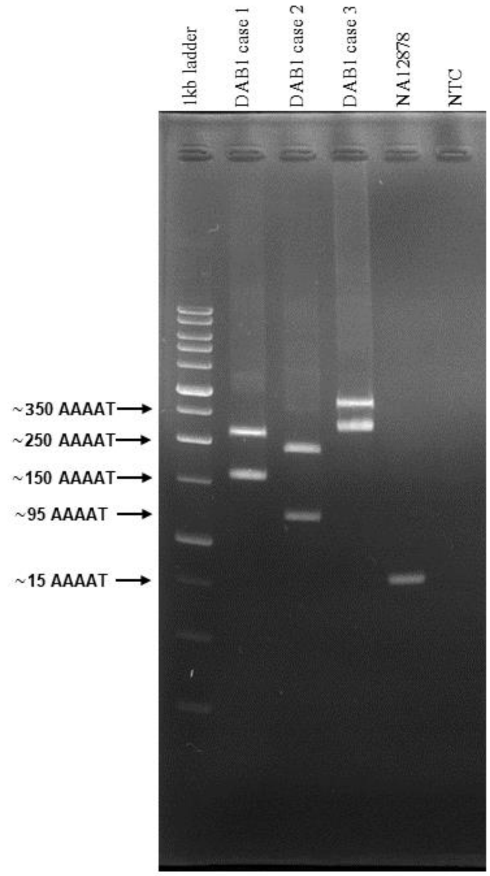

Supplementary Figure S5. A known tandem DNA repeat in *DAB1* is expanded in three unrelated individuals with schizophrenia. The gel electrophoresis shows that three samples (*DAB1* case 1, *DAB1* case 2 and *DAB1* case 3) have amplified alleles with amplicon size larger than that detected in the control DNA (NA12878). The amplified region corresponds to the locus of known disease-causing TREs in *DAB1* for spinocerebellar ataxia type 37 (SCA37). DNA was extracted from peripheral blood. NTC: No template control.

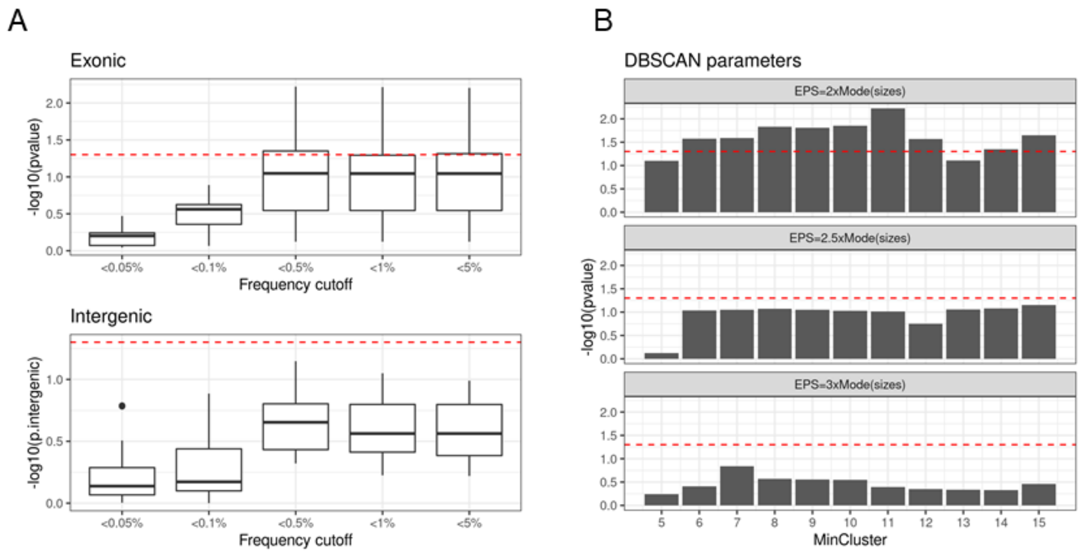

Supplementary Figure S6. DBSCAN parameter and population frequency cut-off optimization.
(**A**) Burden test of exonic (i.e., exon-proximal) and intergenic TREs at different population frequency cut-offs. Data points in box plots were based on different sets of rare TREs identified using 33 different DBSCAN parameter combinations (Methods). (**B**) DBSCAN minimum cluster member and Epsilon parameters optimization using <0.5% as the population frequency cut-off. Bars indicate p-value of the test of rare exonic tandem repeats burden. Dashed red line represents p=0.05. Raw data are provided in Supplemental Table S8.

References

1. Richards S. et al. *Genet Med* **17**, 405-24 (2015).
2. Kearney HM. et a. *Genet Med* **13**, 680-5 (2011).
3. Mojarad BA. et al. *Transl Psychiatry* **11**, 84 (2021).
4. Trost B. et al. *Nature* **586**, 80-86 (2020).
5. Perini GI. et al. *Biol Psychiatry*. **46**, 425-31 (1999).
6. Sistiaga A. et al. *Psychol Med*. **40**, 487-95 (2010).
7. Kobayakawa M. et al. *Neurosci Res*. **72**, 341-6 (2012).
8. Labayru G. et al. *PLoS One*. **13**, e0204227 (2018).
9. Hernández-Hernández O. et al. *Brain*. **136**, 957-70 (2013).
10. Gall-Duncan T, et al. *Genome Res*. **32**, 1-27 (2022).
